# Pepsin and the Lung—Exploring the Relationship between Micro-Aspiration and Respiratory Manifestations of Gastroesophageal Reflux Disease

**DOI:** 10.3390/jpm12081296

**Published:** 2022-08-07

**Authors:** Diana-Elena Iov, Oana-Bogdana Bărboi, Mariana Floria, Andrei Neamțu, Radu Iliescu, Vasile-Liviu Drug

**Affiliations:** 1 Internal Medicine Department, Grigore T. Popa University of Medicine and Pharmacy, 700111 Iași, Romania; 2 Saint Spiridon Emergency Hospital, 700111 Iași, Romania; 3 Physiology Department, Grigore T. Popa University of Medicine and Pharmacy, 700115 Iași, Romania; 4 Pharmacology Department, Grigore T. Popa University of Medicine and Pharmacy, 700115 Iași, Romania

**Keywords:** GERD, pepsin, bronchial asthma, COPD, chronic cough, lung transplant

## Abstract

Gastroesophageal reflux disease (GERD) is one of the most commonly encountered disorders in clinical practice nowadays, with an increasing burden on healthcare systems worldwide. GERD-related respiratory symptoms such as unexplained chronic cough, bronchial asthma or chronic obstructive pulmonary disease (COPD) with frequent exacerbations often pose diagnostic and therapeutic challenges and may require a multidisciplinary approach. Moreover, a potential role of GERD as a risk factor has been proposed for chronic rejection in patients who underwent lung transplantation. Pepsin has gained considerable attention from the scientific community in the last few years as a possible surrogate biomarker for GERD. The aim of this narrative review was to provide an overview of the potential utility of pepsin detection as a marker of micro-aspiration in various biological fluids retrieved from patients with suspected GERD-induced respiratory manifestations and in lung transplant patients with allograft dysfunction. Data on the subject remains highly contradictory, and while certain studies support its applicability in investigating atypical GERD manifestations, at the moment, it would be realistic to accept a modest utility at best. A major lack of consensus persists regarding topics such as the optimal timeframe for fluid collection and cut-off values. Further research is warranted in order to address these issues.

## 1. Introduction

Gastroesophageal reflux disease (GERD) is one of the most commonly encountered disorders in clinical practice nowadays, with an increasing burden on healthcare systems worldwide [1]. It is caused by the retrograde movement of gastric contents back into the oesophagus, which may cause bothersome symptoms or complications and have a notable impact on the quality of life, especially given the significant increase in the proportion of younger patients affected by it [2,3].

In addition to the classic symptoms of heartburn and regurgitation, a wide range of atypical and extra-digestive manifestations have been linked to GERD. GERD-related respiratory symptoms such as unexplained chronic cough, bronchial asthma or chronic obstructive pulmonary disease (COPD) with frequent exacerbations often pose diagnostic and therapeutic challenges and may require a multidisciplinary approach. Moreover, a potential role of GERD as a risk factor has also been proposed for chronic rejection in patients who underwent lung transplantation.

Physiologically, oesophageal mucosal injury and other GERD-related symptoms are prevented by protective mechanisms, such as the proper function of the upper and lower oesophageal sphincters, adequate volume clearance by oesophageal peristalsis, and the esophago-glottic closure reflex. Any disturbances in the structure or function of these protective mechanisms may represent contributing factors in the pathophysiology of GERD-induced oesophageal lesions and extra-digestive manifestations [4]. The retrograde movement of gastric contents into the oesophagus can occur by transient lower oesophageal sphincter (LES) relaxations (TLESR), low LES pressure, swallow-induced LES relaxation, and increases in intra-abdominal pressure during periods with low LES pressure [5]. Evidence suggests that in healthy subjects and GERD patients with no hiatus hernia, the most frequently occurring mechanism is represented by TLESR, which is responsible for up to 90% of reflux episodes [5]. These transient relaxations arise independently of swallowing, with no accompanying oesophageal peristalsis, and persist for a longer period compared to swallowing-associated counterparts [5].

In order to explain the occurrence of GERD-induced respiratory manifestations, two main pathways have been hypothesised. First is the reflux theory, which incriminates the micro-aspiration of gastric contents as the main deleterious event. This may induce respiratory symptoms by causing direct damage to the airways and pulmonary parenchyma. The second proposed mechanism is the reflex theory, which states that acid reflux into the distal oesophagus may determine bronchoconstriction via the vagally-mediated esophago-bronchial reflex [4,6]. In regards to the former mechanism, there has been significant interest in the recent literature surrounding the potential utility of pepsin detection in various biological fluids as a surrogate biomarker for gastroesophageal reflux. 

Pepsin is one of the main components of gastric refluxate, secreted by chief gastric cells as the zymogen pepsinogen, which is then further activated to pepsin in the acidic environment created by the hydrochloric acid in the stomach. Pepsinogen has seven isoforms, which have been classified into two main types: pepsinogen C, which can be found in various tissues and organs (including the lung, where it is produced by type 2 pneumocytes for surfactant processing), and pepsinogen A, which is believed to be produced exclusively in the stomach by gastric chief cells [7,8]. However, this concept has been challenged recently by a study by Rao et al., which detected the presence of pepsinogen A/pepsin A in various tissues and internal organs, in addition to the digestive tract and upper airways (where gastric fluid may have been present by reflux). Therefore, cautious interpretation of pepsin positivity is advised in establishing an extra-oesophageal GERD diagnosis, given this probable baseline expression of pepsinogen A/pepsin A in various other organs. Ideally, a definite positive cut-off value should be investigated in order to clearly establish true positive results and avoid overdiagnosis [8].

The aim of this narrative review was to provide an overview of the potential utility of pepsin detection in various biological fluids retrieved from patients with suspected GERD-induced respiratory manifestations, such as bronchial asthma, COPD and chronic cough. In addition, we also reviewed the available literature on pepsin as a marker of micro-aspiration in lung transplant patients with allograft dysfunction.

## 2. Effects of Pepsin on the Airways

GERD is associated with a wide range of chronic respiratory diseases, and many authors have hypothesised its role in airway remodelling. Damage to the respiratory tract is thought to be caused not solely by the acidic nature of the refluxate but also due to the digestive enzymes it contains, such as pepsin [9]. Recent studies have explored the possibility that pepsin may be more than just a biomarker of micro-aspiration but could actually have damaging proinflammatory effects on the respiratory tract epithelium [10].

Studies on human bronchial epithelial cell cultures experimentally simulated the effect of gastric fluid aspiration by exposing cell cultures to various acidic pH levels and pepsin [9,11]. These studies found that pepsin has cytotoxic properties and promotes inflammation by increasing the release of interleukin (IL)-6 and IL-8, therefore triggering neutrophil recruitment and production of acute phase proteins. It also causes disruption of epithelial integrity, with the most significant damage occurring in the most acidic conditions, suggesting a synergistic negative effect of prolonged exposure to pepsin and acid, possibly due to optimal activation of pepsin at lower pH levels [11,12,13]. It is believed that the degradation of epithelial integrity in combination with these potent chemoattractants may stimulate transendothelial migration of neutrophils into the alveolar space [11,14]. Once there, a range of proteases and toxic metabolites with deleterious effects on cellular membrane integrity are released [15]. These findings are confirmed by data from a study conducted on children with gastroesophageal reflux and persistent asthma-like symptoms, which demonstrated the presence of airway neutrophilic inflammation. Moreover, it demonstrated a positive correlation between neutrophil counts, IL-8 and myeloperoxidase levels, which supports the role of IL-8 in the recruitment and activation of neutrophils, with the subsequent release of proteolytic enzymes in the airways [16]. 

In the early phase, aspiration of gastric contents causes alveolitis, which is subsequently followed by gradually increasing degrees of fibrosis. Markedly higher levels of fibrosis-promoting cytokines such as transforming growth factor β (TGF-β) and connective tissue growth factor (CTGF), along with increased deposition of type I collagen in the pulmonary interstitium and extracellular matrix structure disorders, support these findings [12]. Notably, the essential role that pepsin may play in inducing these pathological changes in the airway epithelium is also supported by an animal study by Fekri et al., which found that exposure to pepsin irrespective of acid can also induce significant inflammation and fibrosis in the respiratory tract of rats [17].

In cases of respiratory manifestations thought to be secondary to GERD, acid-suppressive treatment with proton pump inhibitors (PPIs) is often recommended. Even though they can reduce the acidity and volume of gastric contents, these medications do not eliminate the possibility of weakly acidic and non-acidic reflux. Two in vitro studies investigated the effect of gastric juice retrieved from paediatric patients with and without PPI therapy [11,18]. Interestingly, while both studies found that the gastric contents of patients on acid-suppressive treatment were able to elicit more significant bronchial inflammation when compared to counterparts without PPI therapy, the proposed mechanisms differ. Mertens et al. found no correlation between the severity of inflammation and pepsin or bile acid concentrations [18]. Their research suggests that the bronchial inflammation induced by the gastric juice from patients on PPIs could be due to an increased concentration of bacterial products (i.e., endotoxins) in less acidic gastric environments. On the other hand, Hurley et al. found a statistically significant association between the rise in pepsin levels and the ability of gastric fluid to induce transepithelial migration and airway barrier disruption in patients on PPIs [11]. This hints at the possibility that even though lowering gastric acidity may present some advantages, potentially increasing pepsin levels could actually prove more harmful to the airway epithelium. 

Further research on the effects of pepsin on the respiratory tract in non-acidic conditions found that it could induce mucus hypersecretion in both upper and lower airway epithelial cells [19]. This effect was due to an increase in mucin 5AC expression after exposure to pepsin via the matrix metalloproteinase 9 (MMP9) and phosphorylated nuclear factor κB (NF-κB) pathways. Given their potential involvement in non-acidic reflux-related respiratory manifestations, this research suggests they could be viewed as potential targets for pharmacological intervention in the future. 

**Figure 1:**
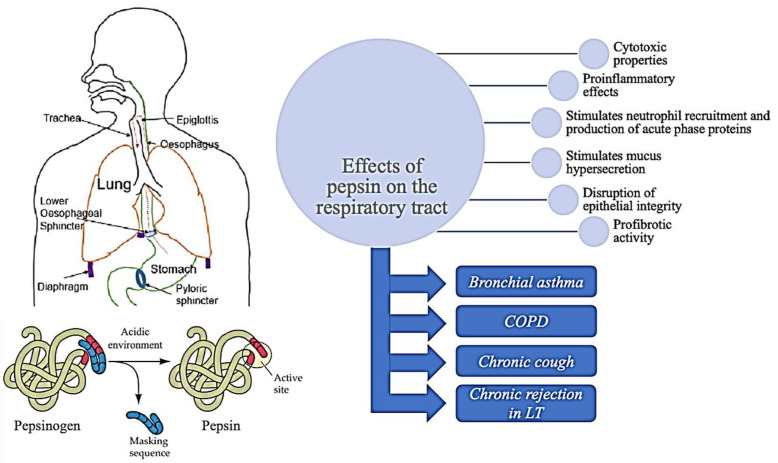
Overview of the reflux theory, summarizing the effects of pepsin on the respiratory tract (COPD = chronic obstructive pulmonary disease; LT = lung transplantation).

An overview of the reflux theory, summarizing the effects of pepsin on the respiratory tract, is presented in Figure 1.

## 3. Pepsin Detection in Bronchial Asthma

There has been increasing interest in the potential utility of detecting and measuring pepsin levels in various biologic fluids retrieved from patients with respiratory manifestations suspected to be due to GERD, including bronchial asthma. An association was observed between GERD and asthma symptoms, as well as frequent exacerbations and poor symptom control [20,21]. However, in spite of the reported association between the two clinical entities, this does not imply causality, and an important controversy still persists regarding this topic. For example, the presence of GERD does not seem to have a significant impact on pulmonary function parameters, and recent studies recognise little to no significant clinical improvement after PPI therapy in bronchial asthma patients with concomitant GERD [22,23,24].

Emilsson et al. found significantly higher levels of pepsin and inflammatory biomarkers in the exhaled breath condensates (EBC) of asthmatic patients with nocturnal GERD when compared to controls [20]. Additionally, an association between nocturnal gastroesophageal reflux and asthma symptoms and exacerbations of respiratory symptoms was observed. Similarly, a cross-sectional study by Timms et al. demonstrated that pepsin levels were higher in EBC samples retrieved from asthmatic patients with GERD compared to their counterparts without a diagnosis of GERD [25]. However, this difference did not reach statistical significance. 

On the other hand, a study by Marshall et al. performed on 25 asthmatic patients showed no significant correlation between salivary pepsin levels and clinical measures of asthma severity [26]. However, one of the major drawbacks of salivary pepsin assessment is the current lack of an optimal timeframe for sample collection. The authors suggest that pooled saliva samples retrieved over a 24-h period may increase the sensitivity of the method, thus improving its diagnostic utility in patients with respiratory manifestations.

Pepsin levels have also been measured from the broncho-alveolar lavage (BAL) fluid collected from asthmatic individuals. Even though pepsin was present in the airways of asthma patients and may represent a marker of aspiration, a correlation between its presence and disease severity could not be demonstrated [27,28]. These results suggest that either BAL pepsin in itself is not an accurate biomarker for respiratory disease, or chronic exposure to even low levels of aspirate might induce inflammatory responses of varying clinical severity. 

When it comes to the paediatric population, a study by Soyer et al., which included asthmatic patients, found that EBC pepsin levels were below the level of detection [29], with the authors suggesting that a more sensitive method with a lower threshold might be necessary in order to evaluate pepsin concentrations in children. In stark contrast to these findings, in the study of Abdallah et al., BAL pepsin positivity was detected both in wheezy infants and in healthy controls, which could imply that the sole presence of pepsin in BAL fluid may not automatically represent a pathological finding [7]. The only significant positive correlations observed in this study were between BAL fluid pepsin levels and mean acid clearance time and duration of the longest acid reflux episode in patients older than one year. However, there was no relationship between pepsin positivity and standard GERD diagnostic testing, such as combined MII-pH monitoring or upper digestive endoscopy.

A summary of the studies investigating pepsin levels in biological fluids retrieved from adult and paediatric subjects and the relationship between pepsin and obstructive lung disease, focusing on bronchial asthma, is presented in Table 1.

## 4. Pepsin Detection in COPD

Associations between GERD and a higher frequency of COPD exacerbations, increased drug usage and subsequent hospitalizations have been described in the literature [30,31,32,33,34]. Pomari et al. assessed the presence of pepsin in the BAL fluid of 42 individuals who required a diagnostic bronchoscopy, all of them with a history of at least one bronchial exacerbation in the previous 12 months [35]. In patients with bronchial asthma and COPD, pepsin was present in all BAL samples, thus supporting the hypothesis that in these patients, GERD could be a contributing factor to the persistence of respiratory symptoms.

A study on patients with obstructive lung diseases by Timms et al. demonstrated that EBC pepsin concentrations in COPD patients with questionnaire-diagnosed GERD were significantly higher when compared both to patients with COPD only and to healthy controls [25]. 

A recent descriptive cross-sectional study by Hashemi-Bajgani et al. performed on 52 COPD patients found a higher mean level of pepsin in BAL fluid retrieved from individuals with a history of COPD exacerbations [36]. However, this difference was not statistically significant, and no correlation between pepsin concentrations and COPD severity or the number of exacerbations could be implied from this study. 

Starosta et al. investigated the potential role of pepsin in BAL fluid as a marker of aspiration in a paediatric population with various chronic lung diseases, comprised mainly of chronic bronchitis patients [37]. They found that pepsin levels correlate positively with the number of extensive proximal reflux episodes but with relatively low specificity.

An observational study by Lee et al. found that pepsin could be detected in 33% of the sputum samples retrieved from clinically stable COPD patients, irrespective of a formal GERD diagnosis [38]. However, neither the presence of pepsin in the sputum nor a diagnosis of GERD confirmed by ambulatory 24-h oesophageal pH monitoring correlated with lung disease severity. Notably, a possible explanation for these findings could reside in the fact that even short isolated reflux episodes, which may have been insufficient to alter the criteria used for defining GERD in this study, could still result in occult aspiration.

In another study that analysed EBC samples retrieved from 10 stable COPD patients, pepsin levels were found to be higher in chronic lung disease patients compared to controls; however, this was in the absence of a relationship to a GERD diagnosis or to markers of proximal or distal reflux [39]. In addition to the aforementioned explanation, the small number of subjects included in the study could also represent an important limitation of this study. 

A summary of the studies investigating pepsin levels in biological fluids retrieved from adult and paediatric subjects and the relationship between pepsin and COPD and chronic bronchitis symptoms is presented in Table 2.

## 5. Pepsin Detection in Chronic Cough

Even though the Montreal consensus recognises chronic cough as an extra-digestive manifestation of GERD [3], a causal relationship is especially challenging to prove. In a landmark study that relied on 24-h ambulatory impedance-pH recording and concurrent acoustic monitoring in order to correctly assess coughing episodes, a temporal association was observed between reflux and cough [40]. Interestingly, given the fact that cough preceded reflux with a similar frequency, the study also hints at the possible existence of a self-perpetuating vicious cycle between the two clinical entities. 

Decalmer et al. reached a similar conclusion in a study on 100 chronic cough patients, observing that coughing frequency is directly related to the total number of reflux episodes, which were mostly limited to the distal oesophagus [41]. Given the fact that the number of proximal reflux events and pepsin concentrations in the sputum or BAL fluid were similar to healthy controls, the authors favour an esophago-bronchial neurogenic reflex as the explanation for this temporal association. This hypothesis is also supported by the findings of a study by Grabowski et al., which revealed that similar levels of pepsin and bile acids were present in the induced sputum retrieved from chronic cough patients and healthy controls [42]. In addition, research has shown that pepsin levels in sputum are inversely correlated with cough frequency, which suggests a protective role of enhanced cough against aspiration of pepsin in the large airways [41]. Results from a paediatric study by Dy et al. seem to support this hypothesis, as levels of salivary pepsin were significantly lower in children with a history of daily chronic cough compared to those presenting without cough [43].

In contrast to these findings, results from the pioneer study, which assessed pepsin levels in the BAL fluid collected from chronic cough paediatric patients, incriminate GERD-related micro-aspiration, thus supporting the reflux theory. In their research, Farrell et al. found significantly increased concentrations of pepsin in chronic cough subjects compared to negative controls, while this difference was not observed in GERD patients without chronic cough [44]. 

Additional evidence supporting this pathogenic mechanism comes from a real-life setting cohort study by Strugala et al. [45]. Their research evaluated pepsin levels in saliva and sputum samples retrieved from 93 chronic cough patients either during daily activities or following three spontaneous coughing episodes. Chronic cough subjects had significantly greater pepsin detection rates and concentrations when compared to control subjects (noteworthy, however, is the fact that control samples were collected from the healthy volunteers at different timepoints—in the morning and 1 h after lunch and dinner).

It is important to mention the fact that although GERD is considered to be one of the main contributing factors to chronic cough in adults, it is a much less common cause in the paediatric population [46]. Martin et al. performed a retrospective study to assess the possible relationship between BAL fluid pepsin positivity and various endoscopic and serologic characteristics in children presenting with chronic cough [47]. In these patients, no correlation was found between the detection of pepsin and results of pH-impedance studies or upper digestive endoscopy findings. However, a significant association was found between pepsin positivity in BAL fluid and viral PCR, hinting at the possibility of an inverse relationship, that of cough-induced gastroesophageal reflux. 

A summary of the studies investigating pepsin levels in biological fluids retrieved from adult and paediatric subjects and the relationship between pepsin and chronic cough is presented in Table 3.

## 6. Pepsin Detection in Lung Transplant Patients

The potential role of GERD as a cause of non-allogenic injury and a risk factor for chronic rejection in patients who underwent lung transplantation (LT) has been hypothesised and investigated in recent literature. The clinical utility of clearly establishing its involvement lies in the fact that it is a modifiable risk factor, with a generous array of potential treatment options, including fundoplication.

Blondeau et al. found that in LT patients, aspiration of gastric contents occurs frequently, as indicated by the presence of pepsin in all BAL fluid samples retrieved from the individuals included in their study [48]. However, pepsin concentrations did not correlate with the occurrence of bronchiolitis obliterans syndrome (BOS) compared with stable LT patients. This suggests that while a possible role of pepsin detection in the airways as a general marker of aspiration could be implied, it is not possible to identify a potential susceptibility to the development of BOS in LT individuals.

The results of another study by Fisichella et al. point to a similar conclusion that pepsin detection in BAL fluid collected from LT patients could indeed demonstrate aspiration [49]. They also found that in individuals in which surgical correction of GERD was performed by laparoscopic Nissen fundoplication, pepsin concentrations were significantly lower compared to LT patients who did not undergo this procedure. In contrast to the findings of Blondeau et al., this study observed a correlation between higher levels of pepsin and increased severity of rejection episodes. In addition to that, they also showed that the detection of pepsin in BAL fluid is associated with a more rapid progression to BOS; this could be explained by the link between multiple episodes of acute rejection and BOS occurrence. 

The same team went on to further investigate BAL fluid cellularity and composition in immune mediators in 105 LT patients in order to identify specific patterns of dysregulation [50]. They found that LT cases with detectable pepsin levels in BAL fluid showed significantly increased neutrophilia and reduced concentrations of IL-12, which was associated with more severe acute cellular rejection. Moreover, pepsin concentrations were found to be significantly increased in patients with BOS.

In a study performed on a smaller population of only 18 LT patients, Griffin et al. aimed to prospectively assess GERD and reflux in the first month after transplantation [51]. Pepsin was detected in most of the BAL fluid samples, suggesting that aspiration can occur frequently and early in the post-transplant period. In addition, proximal reflux events correlated with the BAL fluid neutrophil percentage, which, as mentioned previously, was found to be linked to allograft dysfunction.

Render et al. investigated the utility of pepsin detection in BAL fluid samples collected from LT patients in order to identify those with GERD-related aspirations [52]. Their results showed a 60% sensitivity and 45% specificity of pepsin in BAL fluid when compared to pH monitoring, which rendered it rather inaccurate as a sole predictor of GERD status. However, one of the major limitations of this study is the fact that less than half of the subjects underwent pH monitoring, which could have resulted in significant variability and selection bias. All in all, the evaluation of pepsin in BAL fluid could provide additional data for identifying LT patients who may benefit from surgical treatment of GERD by fundoplication. 

A summary of the studies investigating pepsin levels in biological fluids retrieved from adult patients who underwent lung transplantation is presented in Table 4.

Of important note, it should be stated that some studies also demonstrated the presence of pepsin in various biological fluids collected from healthy individuals. For example, in a study that compared chronic cough patients to controls, pepsin BAL fluid positivity percentage and concentrations were similar between the two groups [41]. In a similar study by Grabowski et al., the pepsin positivity percentage was actually higher in the control group, with no significant difference in pepsin concentrations [42]. Therefore, caution is recommended when interpreting data on the subject, as many studies still yield inconsistent results. 

## 7. Conclusions

Pepsin has gained considerable attention from the scientific community in the last few years as a possible surrogate biomarker for GERD. Many studies have aimed to investigate the potential utility of pepsin detection in individuals suffering from possibly GERD-induced respiratory manifestations, such as chronic cough, bronchial asthma and frequent-exacerbator phenotype COPD. In addition to that, a distinct category of patients is represented by those who underwent lung transplantation and in whom GERD may play a contributing role to chronic rejection. Taking into consideration all recent publications, it is obvious that data on the subject remain highly contradictory, and no clear conclusion can be drawn on the current usefulness of pepsin detection. While certain studies support its applicability in investigating atypical GERD manifestations, at the moment, it would be realistic to accept a modest utility at best. A major lack of consensus still persists regarding essential topics such as the optimal timeframe for biological fluid collection (especially when the focus is placed on salivary pepsin) and specific cut-off values. Further research is warranted in order to address these issues.

## Figures and Tables

**Table 1:** Summary of the studies investigating pepsin levels in biological fluids retrieved from adult and paediatric subjects and the relationship between pepsin and obstructive lung disease (bronchial asthma, COPD).

	Subjects	Age Range	Pepsin Method of Detection and Values	Conclusions
**Abdallah et al. (2016)** [7]	Wheezy infants and healthy controls	3–24 months	BAL fluid pepsin was measured using the Human Pepsin enzyme-linked immunosorbent assay Kit of Glory Science Co., Ltd.: Wheezy infants with silent reflux and wheezy infants with typical GERD symptoms but normal combined MII-pH had significantly higher pepsin levels compared to healthy controls (45.3 ± 8.6 and 42.8 ± 8 versus 29 ± 2.6, *p* < 0.0001 and 0.011, respectively). Wheezy infants with typical GERD symptoms and/or abnormal combined MII-pH had significantly higher pepsin levels compared to healthy controls but not to those with no symptoms and normal impedance-pH (42.1 ± 8.3 versus 29 ± 2.6 and 37.9 ± 6.8, *p* < 0.0001 and >0.05, respectively)	BAL fluid pepsin was positive both in wheezy infants and in healthy controls. The relationship between GERD standard diagnostic tests and BAL fluid pepsin did not reach statistical significance. BAL fluid pepsin showed a statistically significant positive correlation with the mean acid clearance time and the duration of the longest acid episode in infants older than one year.
**Emilsson et al. (2016)** [20]	Patients with nocturnal GERD (nGERD) and age and gender-matched controls	GERD patients: 55.8 ± 6.7 years No GERD: 56.4 ± 7.0 years	Pepsin measured in EBC by ELISA (Wuhan EIAAB Science Co., Ltd., Wuhan, China). In patients without GERD, mean value 0.8 ng/mL (0.8–3.6 ng/mL) In nGERD patients, mean value 2.5 ng/mL (0.8–5.8 ng/mL)	Bronchial asthma and bronchitis symptoms, as well as respiratory exacerbations, were associated with nGERD. Pepsin levels were significantly higher in nGERD patients compared to controls (*p* = 0.03).
**Timms et al. (2012) [25]**	Bronchial asthma and COPD patients and healthy controls	Between 38.00 ± 6.34 years in asthma patients and 70.63 ± 3.391 years in COPD patients	Pepsin measured in EBC by using an in-house quantitative ELISA based on a monospecific antibody to porcine pepsin. Pepsin in subjects with obstructive lung disease and GERD, 9.81 ± IQR 4.38 ng/mL, compared to those without GERD, 4.6 ± IQR 6.95 ng/mL Pepsin in healthy controls, 3.44 ± IQR 7.87 ng/mL COPD group with GERD had a significantly higher median pepsin concentration (11.59 ± IQR 6.86 ng/mL) compared to the COPD control group (3.85 ± IQR 5.71 ng/mL)	Participants with obstructive lung disease and GERD had significantly higher levels of pepsin compared to those without GERD (*p* < 0.002) and healthy controls (*p* = < 0.013). COPD group with GERD had a significantly higher median pepsin concentration than the COPD control group (*p* = 0.0175). Patients with asthma and GERD had a higher mean pepsin concentration compared to the asthma control group but without reaching statistical significance.
**Marshall et al. (2019)** [26]	Bronchial asthma patients	58.7 ± 11.3 years	Pepsin was measured in three saliva samples (a throat-clearing saliva sample provided during a routine clinic visit, half of which was mixed with citric acid, and another sample upon waking, prior to brushing their teeth, drinking or eating in the morning the day after their clinic visit) by non-competitive indirect sandwich ELISA: Present in at least one of the three samples in 56% of patients (mean ± SD = 66.7 ± 76.5 ng/mL)	No significant associations were found between pepsin and clinical measures of asthma severity.
**Rosen et al. (2012)** [27]	Bronchial asthma and chronic cough paediatric patients	67 ± 43 months	Pepsin was analysed in BAL fluid by ELISA using rabbit anti-pepsin antibody diluted and mouse anti-b-actin antibody (CP01, EMD Chemicals, Gibbstown, NJ, USA): Concentration in specimens in which it was detected (44%): mean ± SD = 80.3 ± 87.5 ng/mL	A significantly higher mean lipid-laden macrophage index was found in patients that were pepsin positive compared to pepsin-negative patients (81 ± 54 vs. 47 ± 26, *p* = 0.001).
**Hunt et al. (2017) [28]**	Bronchial asthma patients		BAL fluid pepsin was analysed by using a locally developed indirect ELISA—the primary antibody was specific to porcine pepsin (Biodesign International Cat no W59117G), and the secondary antibody was horse radish peroxidase-conjugated rabbit, anti-goat (Sigma): BAL fluid pepsin detectable in 58.9% of subjects Median concentration of 3.58 ng/mL No significant differences in pepsin levels across the groups when divided by exacerbation frequency (3.27 ng/mL, 2.53 ng/mL and 3.51 ng/mL, respectively)	No significant associations between pepsin level and measures of disease severity asthma control, FEV1, ACQ or exacerbation frequency.
**Soyer et al. (2013) [29]**	Paediatric patients with a presumptive diagnosis of GERD with recurrent respiratory and/or gastrointestinal problems	2–14 years	Pepsin concentrations in EBC specimens were analysed by using homemade indirect ELISA. Concentrations were below the level of detection.	A more sensitive ELISA with a lower threshold of detection may be useful in order to investigate pepsin levels in paediatric populations.

BAL: bronchoalveolar lavage; COPD: chronic obstructive pulmonary disease; EBC: exhaled breath condensate; ELISA: enzyme-linked immunosorbent assay; GERD: gastroesophageal reflux disease.

**Table 2:** Summary of the studies investigating pepsin levels in biological fluids retrieved from adult and paediatric subjects and the relationship between pepsin and COPD and chronic bronchitis symptoms.

	Subjects		Age Range	Pepsin Method of Detection and Values	Conclusions
**Pomari et al. (2016)** [35]	Patients with a history of a chronic cough, plug or dyspnea and abnormal lung examination enrolled for the worsening of respiratory symptoms in spite of regular treatment		20–84 years	Semiquantitative assessment of BAL fluid pepsin using Pep-test (PeptestTM, RD Biomed Ltd., Cottingham, UK). Pepsin was positive in 88% of specimens	A strong positive statistical correlation was found between pepsin detection in bronchial secretions and radiological signs of GERD and GERD diagnosis.
**Hashemi-Bajgani et al. (2019)** [36]	COPD patients with and without exacerbations		COPD group with exacerbation history: 60.88 ± 8.10 years COPD group without exacerbation history: 60.15 ± 9.53 years	BAL fluid pepsin was measured using the standard pepsin kit (ELISA Kit for pepsin) from the USCNK Company. Mean pepsin levels in the group without exacerbations: 118.46 ± 15.44 ng/mL Mean pepsin levels in the group with a history of exacerbations: 107.88 ± 10.7 ng/mL	No association was found between disease severity and the number of exacerbations with micro-aspiration of pepsin.
**Starosta et al. (2007)** [37]	Paediatric patients with chronic bronchitis, allergic asthma, recurrent pneumonia, bronchiectasis, tracheomalacia, primary ciliary dyskinesia and bronchiolitis obliterans	4.7 (3.3–7.5) years	Levels of pepsin in BAL fluid were determined by using a modification of the proteolytic enzyme assay method.	The average concentration of pepsin was higher in the group with extensive proximal acidic gastroesophageal reflux index than in children with reflux index < 2% (*p* < 0.037). Pepsin in BAL fluid of this paediatric group with chronic respiratory symptoms correlated positively with the number of proximal reflux events; however, it does not differentiate patients with reflux from those without.	
**Lee et al. (2014)** [38]	Patients with COPD or bronchiectasis and healthy controls		COPD group: 67.7 ± 7.7 years Bronchiectasis group: 53.7 ± 14.0 years Controls: 36.6 ± 15.1 years	Pepsin levels in sputum samples were analysed using a locally developed ELISA based on a monospecific antibody to porcine pepsin. Samples were collected at four intervals over 24 h: upon waking, mid-morning, mid-afternoon and prior to sleeping (prior to or 1 h after meals). Median pepsin concentration in sputum in the COPD group was 2.84 ± IQR 4.05 ng/mL In bronchiectasis patients, the sputum pepsin concentration was 3.48 ± IQR 4.18 ng/mL	Pepsin in sputum was not related to a diagnosis of GERD. Neither a diagnosis of GERD nor the presence of pepsin in sputum was associated with reduced lung function in COPD or bronchiectasis.
**Lee et al. (2015)** [39]	Patients with COPD or bronchiectasis and healthy controls		COPD group: 58–74 years Bronchiectasis group: 52–69 years Controls: 26–72 years	EBC pepsin concentrations were measured using a locally developed ELISA based on a monospecific antibody to porcine pepsin. EBC pepsin levels in patients with and without GERD in the bronchiectasis group: 3.42 [2.81–3.9] ng/mL vs. 1.97 [0.91–2.73] ng/mL EBC pepsin levels in patients with and without GERD in the COPD group: 2.95 [2.69– 3.73] ng/mL vs. 0.96 [0.41–2.64] ng/mL	A diagnosis of GERD was not associated with a higher concentration of EBC pepsin in bronchiectasis or COPD.

BAL: bronchoalveolar lavage; COPD: chronic obstructive pulmonary disease; EBC: exhaled breath condensate; ELISA: enzyme-linked immunosorbent assay; GERD: gastroesophageal reflux disease.

**Table 3:** Summary of the studies investigating pepsin levels in biological fluids retrieved from adult and paediatric subjects and the relationship between pepsin and chronic cough.

	Subjects	Age Range	Pepsin Method of Detection and Values	Conclusions
**Decalmer et al. (2012)** [41]	Chronic cough patients and healthy controls	Chronic cough group: 55.8 ± 11.0 years Healthy controls: 30–50.8 years	Pepsin was measured in BAL fluid and induced sputum using a plate ELISA based on a monospecific antibody to porcine pepsin. BAL pepsin levels in chronic cough subjects: median 18.2 ng/mL (range, 0–56.4 ng/mL) BAL pepsin levels in control subjects: median 9.25 ng/mL (range, 0–46.9 ng/mL)	Log cough frequency was inversely related to sputum pepsin concentration: subjects with higher cough frequency had lower sputum pepsin concentrations. Therefore, coughing appears to be protective, reducing pepsin concentration in the larger airways of patients with chronic cough.
**Grabowski et al. (2011)** [42]	Chronic cough patients and healthy controls	Chronic cough group: 21–75 years Healthy controls: 24–66 years	Pepsin levels in induced sputum were measured by ELISA kit (USCN Life Science Inc. Wuhan, China). Chronic cough group: pepsin level was 6.4 ± 6.4 ng/mL Healthy controls: pepsin level was 7.3 ± 7.2 ng/mL Pepsin was detectable in 48.8% of samples in CC patients and in 60% of healthy controls	In pepsin-positive samples, no significant difference in pepsin concentration could be found between chronic cough patients and the healthy control group.
**Dy et al. (2016)** [43]	Paediatric patients undergoing testing for the evaluation of GERD	1–19 years	Random saliva samples were collected for pepsin testing. For subjects who were unable to produce a salivary sample, an oropharyngeal saliva aspirate was obtained. All samples were obtained after fasting for at least 2 h. Saliva specimens were analyzed using the PepTest (RD BioMed, Hull UK). 42% of samples were pepsin positive, with median concentration of pepsin in the saliva 10 ng/mL (IQR 10–55) Pepsin concentrations were lower among patients with a recent history of daily chronic cough than those without cough—median (IQR): 0 (0, 10) versus 18 (5, 49)	No significant difference between the pepsin-positive and pepsin-negative groups in terms of distribution of acid, nonacid, total reflux episodes, full column reflux or any other reflux variable. There was no significant correlation between the number of reflux episodes and pepsin concentrations.
**Farrell et al. (2006)** [44]	Children undergoing general anaesthesia as part of their investigations for symptoms of GERD. Positive control group: intubated children with proven macroscopic aspiration. Negative control group—children with no history of GERD or respiratory disease undergoing general anaesthesia for elective surgery.	< 14 years	A BAL fluid pepsin assay was developed based on a sandwich ELISA principle using 2 primary antiporcine pepsin antibodies. Pepsin was detected in the BAL fluid of a significantly greater proportion of subjects in the positive control group compared to the negative control group Median BAL pepsin level was significantly greater in the positive control group compared to the negative control group—median 0 vs. 24.13 ng/mL The sensitivity, specificity, and positive and negative predictive values for the pepsin assay to detect pulmonary aspiration were 80%, 100%, 100%, and 93%, respectively	Pepsin concentrations in the BAL fluid of children with proximal oesophageal GERD were significantly higher in subjects with chronic cough compared to negative controls. No such difference was observed between participants with proximal GERD without cough and negative controls.
**Strugala et al. (2015)** [45]	Chronic cough patients and a previously investigated healthy volunteer population	Chronic cough group: 58.4 ± 13.8 years	Patients were instructed to provide three expectorated saliva/sputum samples during daily activities and immediately after three spontaneous coughing episodes. Pepsin levels were assessed using Peptest (RD Biomed Ltd., Cottingham, UK).	Chronic cough patients had a significantly higher prevalence of pepsin detection (*p* < 0.0001) and increased pepsin concentration compared to the control group.
**Martin et al. (2021)** [47]	Children undergoing flexible bronchoscopy and bronchoalveolar lavage	4.9 (2.2–9.1) years	BAL fluid pepsin was assessed by enzyme-linked immunosorbent gastric pepsin A assay.	No demographic characteristic of our patient population was significantly associated with pepsin positivity. Pepsin-positive groups did not have more severe respiratory symptoms. No significant difference in pulmonary function testing was observed between the two groups. No correlation was found between pepsin detection and pH-impedance or esophago-gastro-duodenoscopy findings. A significant association was observed between pepsin positivity and viral PCR, hinting at the possibility of cough-induced reflux.

BAL: bronchoalveolar lavage; COPD: chronic obstructive pulmonary disease; ELISA: enzyme-linked immunosorbent assay; GERD: gastroesophageal reflux disease; PCR: polymerase chain reaction.

**Table 4:** Summary of the studies investigating pepsin levels in biological fluids retrieved from adult subjects who underwent lung transplantation.

	Subjects	Age Range	Pepsin Method of Detection and Values	Conclusions
**Blondeau et al. (2008)** [48]	Lung transplant recipients BAL fluid samples were collected from non-transplant subjects requiring a bronchoscopy as controls	52 (19–69) years	Pepsin was measured using an ELISA using a primary polyclonal antibody to porcine pepsin and goat immunoglobulin G as a secondary antibody. All transplanted patients had detectable pepsin in their BAL fluid samples The median pepsin concentration in lung transplant recipients was significantly higher compared to non-transplant patients: 541 (187–946) ng/mL, compared to 24 (0–25) ng/mL	Gastric aspiration occurred frequently after lung transplantation, as shown by the presence of pepsin in BAL fluid specimens retrieved from all transplanted patients. Patients with bronchiolitis obliterans syndrome did not have increased GERD and did not have a particularly higher concentration of pepsin in BAL fluid. An increased prevalence of reflux could not be found in these patients compared to stable transplant recipients. No significant correlation was found between reflux and FEV1.
**Fisichella et al. (2011)** [49]	Lung transplant recipients (divided into GERD positive and negative) and healthy controls		BAL fluid pepsin levels were measured by a locally developed ELISA in the laboratories at the Burn and Shock Trauma Institute at Loyola University Medical Center using a monospecific antibody to porcine pepsin (Calbio-chem/EMD4Biosciences, Gibbstown, NJ, USA). Pepsin was undetectable in the BAL fluid samples from controls Pepsin levels were increased in the BAL fluid of lung transplant recipients regardless of their reflux status Pepsin concentrations were significantly lower after surgical correction of reflux (*p* = 0.029)	Subjects with any detectable pepsin concentrations had a quicker progression to bronchiolitis obliterans syndrome than patients with undetectable pepsin (*p* = 0.058) Greater levels of pepsin were associated with worse episodes of rejection.
**Fisichella et al. (2013)** [50]	Lung transplant recipients (divided into bronchiolitis obliterans syndrome positive and negative)	59 years (IQR 50–62 years)	BAL fluid pepsin levels were measured by a locally developed ELISA in the laboratories at the Burn and Shock Trauma Institute at Loyola University Medical Center using a monospecific antibody to porcine pepsin (Calbio-chem/EMD4Biosciences, Gibbstown, NJ, USA). Subjects with bronchiolitis obliterans syndrome had significantly higher BAL fluid pepsin concentrations (*p* = 0.001) Pepsin levels in the bronchiolitis obliterans syndrome-positive group: 31 (0–105) ng/mg Pepsin levels in the bronchiolitis obliterans syndrome-negative group: 2 (0–24) ng/mg	Detectable pepsin concentrations in BAL fluid samples from lung transplant recipients were associated with significantly increased neutrophilia and reduced concentrations of IL-12, which was associated with more severe acute cellular rejection.
**Griffin et al. (2013)** [51]	Lung transplant recipients within the first month post-transplantation A reference stable control range was established in 7 optimally stable patients independent of the study cohort.	22–59 years	An in-house, indirect pepsin ELISA with a specific antipepsin antibody and an antisheep/goat secondary antibody was used to evaluate BAL fluid pepsin levels. Pepsin levels in the study group—median: 18 ng/mL; range: 0–43 ng/mL Pepsin levels in controls—median: 5.5, range: 0–12.6 ng/mL The median from the study population was higher but without reaching statistical significance (*p* = 0.1)	Aspiration and allograft injury may occur in the early posttransplant period.
**Reder et al. (2014)** [52]	Lung transplant recipients	54.4 ± 12.9 years	Pepsin concentrations were measured by ELISA using a monoclonal antibody to porcine pepsin as described by the manufacturer (Biodesign International, Saco, ME, USA). EBC and BAL fluid specimens were retrieved during routine follow-up visits. The sensitivity and specificity of pepsin in BAL were 0.60 and 0.45, respectively The sensitivity and specificity of pepsin in EBC were 0.22 and 0.79, respectively	Typical symptoms of GERD did not predict the presence or absence of pepsin in BAL. Symptoms such as aspiration and bronchitis had greater predictive value.

BAL: bronchoalveolar lavage; COPD: chronic obstructive pulmonary disease; EBC: exhaled breath condensate; ELISA: enzyme-linked immunosorbent assay; GERD: gastroesophageal reflux disease.

## Data Availability

Not applicable.
